# A cross-sectional, population-based study of HIV physicians and outpatient health care use by people with HIV in Ontario

**DOI:** 10.1186/s12913-015-0723-5

**Published:** 2015-02-15

**Authors:** Claire E Kendall, Jenna Wong, Monica Taljaard, Richard H Glazier, William Hogg, Jaime Younger, Douglas G Manuel

**Affiliations:** C.T. Lamont Primary Health Care Research Centre, Bruyère Research Institute, 43 Bruyère St, RM 337Y, Ottawa, Ontario K1N 5C8 Canada; Department of Family Medicine, University of Ottawa, 43 Bruyère St., Floor 3JB, Ottawa, Ontario K1N 5C8 Canada; Department of Epidemiology, Biostatistics, and Occupational Health, McGill, 1020 Pine Ave. West, Montreal, Quebec Canada; Department of Epidemiology and Community Medicine, University of Ottawa, 451 Smyth Rd., Room 3105, Ottawa, Ontario K1H 8M5 Canada; Ottawa Hospital Research Institute, 725 Parkdale Ave., Ottawa, Ontario Canada; Institute for Clinical Evaluative Sciences, 2075 Bayview Ave., Room G1-06, Toronto, Ontario M4N 3M5 Canada; Centre for Research on Inner City Health, Li Ka Shing Knowledge Institute, St. Michael’s Hospital, 30 Bond St., Toronto, Ontario M5B 1W8 Canada; Department of Family and Community Medicine, St. Michael’s Hospital, 30 Bond St., Toronto, Ontario M5B 1W8 Canada; Department of Family and Community Medicine, University of Toronto, 500 University Ave., 5th Floor, Toronto, Ontario M5G 1V7 Canada

**Keywords:** Human immunodeficiency syndrome, Primary health care, Chronic disease, Comorbidity, Multimorbidity, Health services delivery

## Abstract

**Background:**

People with HIV are living longer and their care has shifted towards the prevention and management of comorbidities. However, little is known about who is providing their care. Our objective was to characterize the provision of HIV care in Ontario by physician specialty.

**Methods:**

We conducted a retrospective population-based observational study using linked administrative databases in Ontario, Canada, a single payer health care system. All Ontarians with HIV were identified using a validated case ascertainment algorithm. We examined office-based health care visits for this cohort between April 1, 2009 and March 31, 2012. Physician characteristics were compared between specialty groups. We stratified the frequency and distribution of physician care into three categories: (a) care by physician specialty (family physicians, internal medicine specialists, infectious disease specialists, and other specialists), (b) care based on physician caseload (low, medium or high categorized as ≤5, 6-49 or ≥50 HIV patients per physician), and (c) care that is related to HIV versus unrelated to HIV.

**Results:**

Family physicians were older, graduated earlier, were more often female, and were the only group practicing in rural settings. Unlike other specialists, most family physicians (76.8%) had low-volume caseloads. There were 406,411 outpatient visits made by individuals with HIV; one-third were for HIV care. Family physicians provided the majority of care (53.6% of all visits and 53.9% of HIV visits). Internal medicine specialists provided 4.9% of all visits and 9.6% of HIV visits. Infectious disease specialists provided 12.5% of all visits and 32.7% of HIV visits. Other specialties provided 29.0% of visits; most of these (33.0%) were to psychiatrists.

**Conclusions:**

The distribution of visits to physicians caring for HIV patients reveals different patterns of health care delivery by specialty and HIV caseload. Further research should delineate how specialties share care for this population and how different patterns relate to quality of care.

## Background

People with HIV on combination antiretroviral therapy (ART) are now living longer [1,2]. This increase in HIV survival rates means there is a growing prevalence of people with longstanding HIV in Canada and other high-income countries. With this increased survival, people with HIV are likely to acquire additional chronic conditions due to normal aging as well as the effects of HIV and its treatment [3-5]. In prioritizing care for HIV patients, this has led to a shift away from a specialist focus and towards the prevention and management of comorbidities.

This shift raises questions about how to provide the best care for this diverse and complex population. These questions are particularly relevant in the United States, where the Affordable Care Act has the potential to extend health care coverage for a large number of people living with HIV [6,7]. In the early ART-era, a high-volume specialist approach was the main source of care of this population and resulted in improved HIV-specific outcomes [8-10]. However, there is increasing recognition that specialty HIV providers and those with high HIV caseloads are less comfortable preventing and managing the comorbidities associated with chronic HIV infection [11-15]. A new medical home model consistent with chronic disease management of other conditions that bridges both primary and specialty care is likely required [6,16-18].

Little is known about who is actually providing care for people with HIV. One challenge has been lack of standardization of provider terminology in the literature. A “primary care provider” may be defined based on role (i.e. first point source of care or physician responsible for coordination of care) or based on accredited specialty training (i.e. certified family medicine or general medicine specialist). An HIV “specialist” may be accredited as such through residency training or further accreditation, or self-defined based on volume of HIV care or experience. More information about HIV providers and their provision of care to people with HIV is required for health services planning.

In the US, the majority of accredited HIV “specialists” are male, specialty trained, 79% have an annual caseload of over 200 HIV patients, and about 40% are over 50 years of age [16,17]. A survey of U.S. primary care providers found that only 54% report treating HIV patients [16]. Most of these primary care providers were female, family medicine trained, urban practicing, and 36% reported an annual caseload of over 200 HIV patients [7]. Similar ambiguity exists in Canada, where little is known about who is providing HIV care. In a survey of Canadian family physicians, only 33.4% of respondents reported providing any level of care to people with HIV in 2001 [19]. Another study found that even when patients with HIV had a previously identified family physician, many did not identify with and seek care from that provider [15].

The objectives of this study were to (1) determine the proportion of physician care provided to patients with HIV in Ontario by physician specialty (family physicians, infectious disease specialists, general internists, and others), (2) describe the amount and type of care by physician specialty. The study includes virtually all HIV patients receiving care in Ontario, an ethnically diverse industrialized setting with HIV patients with a range of sociodemographic backgrounds and disease risk factors. The Ontario health care system is a single payer system for physician services, and to our knowledge, this is the first such population-based study.

## Methods

### Study design

We conducted a retrospective observational study to examine the characteristics of and health care visits to physicians caring for people living with HIV in Ontario, Canada. To do this, we analyzed the administrative databases held at the Institute for Clinical Evaluative Sciences (ICES) comprising data on over 13 million individuals from the province of Ontario. These databases are made available to accredited researchers through a data sharing agreement with the Ontario Ministry of Health and Long Term Care and are linked using unique, encoded identifiers and analyzed at ICES in accordance with the provincial Personal Health Information Protection Act. This study was approved by the Ottawa Hospital and Sunnybrook Health Sciences Centre Research Ethics Boards.

### Participants

We identified eligible individuals in Ontario from the Registered Persons Database (RPDB), an electronic registry that contains patient demographic information, including age, sex, postal code and mortality data on all Ontarians eligible for coverage under the provincial health insurance plan. To obtain a cohort of people with HIV in the province, we used data from the Ontario Health Insurance Program (OHIP) billing claims system, which records claims for approximately 95% of physician services conducted in the province. To these data, we applied a previously validated algorithm to people 18 years of age and older and living in Ontario between April 1, 1992 and March 31, 2012 to identify those with HIV [20]. Briefly, this algorithm requires at least 3 physician claims (International Classification of Diseases, Ninth Revision (ICD-9) code for HIV infection (042, 043, 044)) over a 3-year period. It has a sensitivity of 96.2% (95% CI 95.2% - 97.9%) and specificity of 99.6% (95% CI 99.1% - 99.8%) for identifying people with HIV and receiving care in Ontario.

We then used the OHIP database to identify all outpatient health care visits made to physicians by patients in our HIV cohort between April 1, 2009 and March 31, 2012 to derive our physician cohort.

### Main measures

We obtained information about physicians in the cohort (including age, sex, and year of graduation from medical school) from the ICES Physician Database (IPDB). This database comprises information from the Ontario Health Insurance Plan (OHIP) Corporate Provider Database (CPDB), the Ontario Physician Human Resource Data Centre (OPHRDC) database and the OHIP database of physician billings. The CPDB contains information about physician demographics, specialty training and certification, and practice location. This information is validated against the OPHRDC database, which verifies this information through telephone interviews with all physicians practicing in Ontario.

To assign physicians to a rurality category, we used the postal code of the physician’s main practice venue along with the Rurality Index of Ontario [21]. Physicians were categorized as practicing in major urban areas (score 0 to 9), non-major urban areas (10 to 44), or rural areas (45 or higher). We used the number of unique patients in the HIV cohort that a physician billed for at least once during the 3-year study period to determine the physician’s HIV caseload, categorized as low (≤5), medium (6-49), or high-volume (≥50) practice, as 6-49 patients is a HIV volume threshold that may lead to improved care [9]. As patients may have seen more than one physician, the HIV patient population of each physician was not mutually exclusive. If physician specialty was not available in the IPDB database, then the most common specialty code among the physician’s office billings in the OHIP database was used to define their specialty. Specialty was categorized into four groups: as “Family Medicine/General Practice” (comprising the specialties of family medicine, family medicine/emergency medicine, general practice or community medicine, all of whom may be licensed to practice family medicine/general practice in Ontario), “Internal Medicine”, “Infectious Diseases” or “Other”. Finally, using billing diagnoses, we classified each visit as either an HIV visit (any diagnosis code of ‘042’ , ‘043’ , or ‘044’) or non-HIV visit (any non-HIV diagnostic code). In Ontario, only a single diagnostic code can be billed per visit.

### Statistical analysis

We used descriptive statistics to compare the characteristics of providers across the specialty categories using two-sample t-tests (for continuous variables) and chi-squared tests (for categorical variables). We stratified the frequency and proportion of physician care into three categories: (a) care by physician specialty, (b) care based on physician caseload, i.e. the number of HIV patients per physician, and (c) care that is related to HIV versus unrelated to HIV. Frequencies less than or equal to 5 were excluded from analysis by collapsing them with the closest variable category.

All statistical analyses were performed using SAS version 9.2 (SAS Institute, Cary, North Carolina).

## Results

After excluding individuals who were not alive on or not diagnosed with HIV by April 1, 2009, those who had an invalid OHIP number (n = 9,046), and those with an HIV diagnosis date after their date of death (n = 1), there remained 14,282 individuals aged 18 years or older in our HIV cohort. Their characteristics are described in more detail previously [5].

There were 4,756 physicians who provided care to people in the HIV cohort, defined as having submitted at least one claim within the study period for a patient in the HIV cohort: 3,699 (77.8%) family physicians, 55 (1.6%) infectious disease specialists, 70 (1.5%) internal medicine specialists, 895 (18.8%) other specialists, and 30 (0.6%) physicians with no identified specialty.

Table 1 presents the characteristics of physicians by specialty. The ‘other’ specialty physicians represented a diverse group of specialists (Table 2), and were therefore excluded from this comparison of demographic characteristics. There were several demographic differences between the specialties. The mean ages of the internal medicine and infectious disease specialists were significantly lower than that of family physicians (47.0 years and 47.4 years vs. 49.8 years). Family physicians had a higher proportion of female physicians than the other specialty groups (30.8% of family physicians vs. 20.0% of internal medicine specialists and 23.6% of infectious disease specialists), although this difference was not statistically significant. There were also significant differences in the time since graduation between specialties; 58.2% of family physicians graduated prior to 1990, compared with 38.6% of internal medicine specialists and 47.3% of infectious disease specialists.

**Table 1:** **Characteristics of physicians by specialist category**

**Variable**		**All**	**Family medicine**	**Internal medicine specialist**	**Infectious disease specialist**	**p-value**
		**N = 3,824**	**N = 3,699**	**N = 70**	**N = 55**	
Age (years)	Mean ± SD	49.7 ± 11.2	49.8 ± 11.1	47.0 ± 14.1	47.4 ± 9.2	0.033
Age category (years)	<40	1,711 (44.7%)	732 (19.8%)	29 (41.4%)	12 (21.8%)	<0.001
	40-54	986 (25.8%)	1,660 (44.9%)	19 (27.1%)	32 (58.2%)	
	55-64	773 (20.2%)	966 (26.1%)	12 (17.1%)	11 (20%)*	
	>64	347 (9.1%)	334 (9.0%)	10 (14.3%)		
Sex	F	1,165 (30.5%)	1,138 (30.8%)	14 (20.0%)	13 (23.6%)	0.276
Year of graduation	Missing	320 (8.4%)	307 (8.3%)	7 (10.0%)	6 (10.9%)	<0.001
	>2000s	363 (9.5%)	471 (12.7%)	18 (25.7%)	23 (41.8%)*	
	1990s	829 (21.7%)	765 (20.7%)	18 (25.7%)		
	1980s	1,017 (26.6%)	992 (26.8%)	10 (14.3%)	15 (27.3%)	
	1970s	801 (20.9%)	815 (22.0%)	6 (8.6%)	11 (20.0%)*	
	Pre-1970	494 (12.9%)	349 (9.4%)	11 (15.7%)		
Rural status of physician	Missing	114 (3.0%)	112 (3.0%)	<=5	0	0.002
	Major urban	3,092 (80.9%)	2,972 (80.3%)	65 (92.9%)	55 (100.0%)	
	Non-major urban	451 (11.8%)	448 (12.1%)	<=5	0	
	Rural	167 (4.4%)	167 (4.5%)	<=5	0	
Caseload volume	<=5	2,870 (75.1%)	2,841 (76.8%)	25 (35.7%)	20 (36.4%)*	
	6-49	830 (21.7%)	779 (21.1%)	35 (50.0%)		<0.001
	> = 50	124 (3.2%)	79 (2.1%)	10 (14.3%)	35 (63.6%)	

*Some categories are collapsed to avoid reporting cell sizes < =5.

**Table 2:** **Distribution of visits by other specialists**

**Provider specialty**	**Visits (n)**	**Visits (%)**
Psychiatry	38830	33.0
Dermatology	8015	6.8
Ophthalmology	7756	6.6
Obstetrics and gynecology	6823	5.8
General surgery	5919	5.0
Gastroenterology	5434	4.6
Orthopedic surgery	3982	3.4
Urology	3881	3.3
Otolaryngology	3785	3.2
Cardiology	2868	2.4
Medical microbiology	2708	2.3
Anesthesia	2500	2.1
Plastic surgery	2318	2.0
Hematology	2247	1.9
Radiation oncology	2213	1.9
Neurology	2205	1.9
Endocrinology	2166	1.8
Respirology	2021	1.7
Pediatrics	1844	1.6
Nephrology	1842	1.6
Medical oncology	1415	1.2
Rheumatology	1400	1.2
Physical medicine and rehab.	1100	0.9
Emergency medicine	1004	0.9
Vascular surgery	717	0.6
Clinical immunology	625	0.5
Geriatric medicine	416	0.4
Thoracic surgery	327	0.3
Neurosurgery	320	0.3
Diagnostic radiology	290	0.2
Hematological pathology	176	0.1
Anatomical pathology	173	0.1
Cardio. And thoracic surgery	168	0.1
General pathology	86	0.1
Medical biochemistry	35	0.0
Medical genetics	27	0.0
Occupational medicine	22	0.0
Therapeutic radiology	13	0.0
Pediatric cardiology	9	0.0
Nuclear medicine	5	0.0
Total	117685	

There were significant differences in practice location between specialties. In particular, all infectious disease specialists and 92.9% of internal medicine specialists practiced in major urban settings. In contrast, 16.6% of family physicians had practices in rural or non-major urban settings.

There were significant differences between specialties in the distributions of low, medium and high-volume physicians based on their HIV caseloads. The majority of family physicians (76.8%) had low-volume caseloads. Only 2.1% of family physicians had high-volume caseloads. In contrast, the majority of infectious disease specialists (63.6%) had high-volume caseloads. Internal medicine specialists were more evenly distributed, with 50.0% having medium-volume caseloads. Subanalysis revealed that there was little variation in prevalence of rurally-practicing family physicians among those with low, medium, or high HIV caseloads (data not shown).

**Figure 1:**
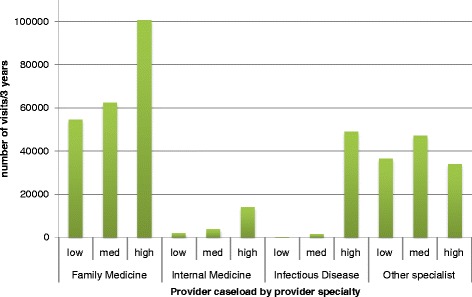
**Number of visits to physicians by physician HIV caseload volume and specialty.** Caseload volume categories by number of patients in HIV cohort seen during study period: low (≤5 patients), medium (6-49 patients) and high (50+ patients).

**Figure 2:**
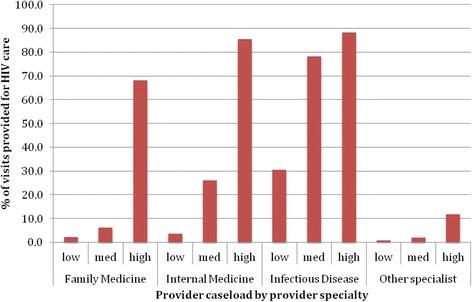
**Proportion of all visits to physicians that are HIV visits by physician HIV caseload volume and specialty.** Caseload volume categories by number of patients in HIV cohort seen during study period: low (≤5 patients), medium (6-49 patients) and high (50+ patients).

The distribution and proportion of outpatient health care visits for our HIV cohort (total visits, HIV visits and non-HIV visits) by specialty category is presented in Table 3, Figures 1 and 2. One third of all visits were for HIV care (33.6% or 136,590 of a total of 406,411 outpatient health care visits).

**Table 3:** **Distribution and proportion of outpatient health care visits between April 1, 2009 and March 31, 2012 (all visits, HIV visits and non-HIV visits) by specialist category and HIV caseload**

**Physician specialty**	**Physician HIV caseload**	**All visits**		**HIV visits**		**Non-HIV visits**		**Proportion HIV visits/all visits**
		**(n)**	**(%)**	**(n)**	**(%)**	**(n)**	**(%)**	**(%)**
Family Medicine	Low	54,712	13.5%	1,246	0.9%	53,466	19.8%	2.3%
	Medium	62,541	15.4%	3,841	2.8%	58,700	21.8%	6.1%
	High	10,0597	24.8%	68,503	50.2%	32,094	11.9%	68.1%
	All	217,850	53.6%	73,590	53.9%	144,260	53.5%	33.8%
Internal Medicine	Low	2,106	0.5%	75	0.1%	2,031	0.8%	3.6%
	Medium	3,872	1.0%	1,007	0.7%	2,865	1.1%	26.0%
	High	14,110	3.5%	12,060	8.8%	2,050	0.8%	85.5%
	All	20,088	4.9%	13,142	9.6%	6,946	2.6%	65.4%
Infectious Disease	Low	118	0.03%	36	0.03%	82	0.03%	30.5%
	Medium	1,632	0.4%	1,276	0.9%	356	0.1%	78.2%
	High	49,038	12.1%	43,316	31.7%	5,722	2.1%	88.3%
	All	50,788	12.5%	44,628	32.7%	6,160	2.3%	87.9%
Other specialist	Low	36,465	9.0%	293	0.2%	36,172	13.4%	0.8%
	Medium	47,239	11.6%	894	0.7%	46,345	17.2%	1.9%
	High	33,981	8.4%	4,043	3.0%	29,938	11.1%	11.9%
	All	117,685	29.0%	5,230	3.8%	112,455	41.7%	4.4%
Total		406,411	100%	136,590	100%	269,821	100%	33.6%

Caseload volume categories by number of patients in HIV cohort seen during the 3-year study period: low (≤5 patients), medium (6-49 patients) and high (50+ patients).

Family physicians provided the majority of outpatient visits for HIV patients in Ontario (217,850 visits, or 53.6% of all visits and 53.9% of HIV visits). Family physicians in low- and medium-volume practice provided 28.9% of all visits, most of which were for non-HIV care (95.7%). In contrast, family physicians in high-volume practice provided 24.8% of all visits, most of which were for HIV care (68.1%).

Internal medicine specialists provided 20,088 visits for HIV patients in Ontario (4.9% of all visits and 9.6% of HIV visits). Most visits were to specialists in high-volume practice (70.2%). For those in low- and medium-volume practice, only 3.6% and 26.0% of visits were HIV visits, respectively. However, for those in high-volume practice, 85.5% of visits were HIV visits.

Infectious disease specialists provided 50,788 visits for HIV patients in Ontario (12.5% of all visits and 32.7% of HIV visits). Again, most visits were to specialists in high-volume practice (96.6%). For those in low-volume practice, only 30.5% of visits were HIV visits. However, for those in medium and high-volume practice, 78.2% and 88.3% were HIV visits.

**Figure 3:**
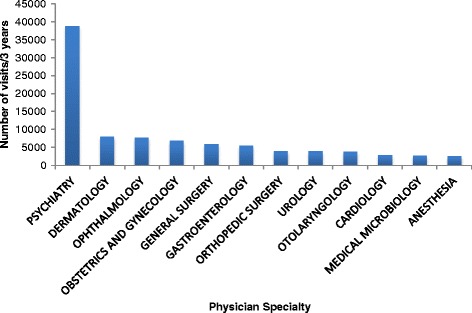
**Distribution of visits by other specialists tables and captions.**

Other specialties provided 117,685 visits to people with HIV in Ontario (29.0% of all visits and 3.8% of HIV visits); only 4.4% of these visits were billed for HIV care. Psychiatrists provided the majority of these visits (33.0% of other specialist visits and 9.6% of all visits). Figure 3 shows the number of visits provided by those specialties for which the proportion of visits exceeded 2% of the total. A complete distribution of the proportions of visits to other specialties is presented in Table 2.

## Discussion

Our study describes two key aspects of HIV care in Ontario. First, we found that family physicians providing care to people with HIV are, on average, more often female, older and graduated longer ago than internal medicine and infectious disease specialists caring for these patients. Family physicians were the only specialty with a presence in rural settings. Contextualizing the respective contribution of physician specialty to HIV care has been difficult, as there has been a lack of standardization of provider terminology in the HIV literature. For example, studies describe ‘HIV primary care’ regardless of the specialty of the physician. As such, this study makes an important contribution to our understanding of the HIV physician workforce.

Second, our study describes how these physicians are providing care. Family physicians, most of whom have low- or medium- HIV caseloads, were by far the most common specialty providing care to people with HIV in Ontario. Furthermore, family physicians provided the majority of both HIV and non-HIV care. Internal medicine and infectious disease specialists with lower HIV caseloads provided fewer and mostly non-HIV visits, but higher caseload specialists provided mostly HIV visits. These findings may speak to patterns of consultation or shared care at lower caseloads versus a specialist acting as the ‘primary care provider’ , measured as the provision of high proportions of all care, at higher HIV caseloads. Finally, other specialties provide a large proportion of care. Psychiatrists provided an amount of care to people with HIV that approaches HIV specialists (9.6 versus 12.5% of all visits).

This study adds to understanding how volume of care and provider specialty are reflected in the actual health care utilization of people living with HIV. Historically, specialty physicians with high HIV caseloads have been required to manage the complexities of HIV care. However, we know that for many complex chronic conditions specialty care may improve disease-specific indicators while strong primary care is required for improved whole-person care for those with multiple conditions [22]. Our findings are consistent with previous work demonstrating a decrease in ambulatory visits to infectious disease specialists associated with a concomitant increase in visits to other physician specialties [23], but visits to family physicians have not been previously explored. Representing 30.2% of the 12,252 Ontario family physicians providing care in 2009, the proportion of family physicians providing care to any patients with HIV is not greatly changed from the 24% reported in a 2001 Canadian family physician survey [19]. However, the proportion of care this represents for this population is surprisingly substantial. Questions remain regarding the best ways to merge HIV expertise and generalist knowledge to meet the needs of this increasingly diverse population [17,24].

Our findings have implications for health care planning, especially given that the Affordable Care Act may substantially increase the number of people with HIV eligible for health care insurance in the United States. Family physicians caring for people with HIV are older and graduated many years ago, yet are the only group practicing in rural settings. These results reflect concerns in the United States of an aging HIV workforce and the need for community-based care [17,24]. In addition, our results highlight the substantial mental health resources required for people with HIV, consistent with the observed high mental health burden in this population.

There are several limitations to our study. First, although we used a highly validated algorithm to identify people with HIV, the algorithm only identified patients who were being cared for by physicians. We were not able to identify those who were unaware of their HIV status (estimated to be 26% of prevalent infections in Canada [25]), or those not accessing health care, who may differ from those in care [26]. As the focus of our study was to describe who is actively providing care to people with HIV, this constraint is appropriate to the research question. Furthermore, there are some practice settings in Ontario (most notably community health centres) whose data were not included in the administrative databases; thus, HIV patients seeking care in these settings could not be included in the study. While community health centres are estimated to serve only 0.9% of the Ontario population [27], they do serve more marginalized populations, thus we may be missing a higher proportion of HIV patients by not capturing care in these settings. Second, we are unable to measure the contribution of care provided by other health care providers who do not bill the provincial insurance system or the care provided by community based HIV/AIDS agencies. Third, as only one billing code is recorded per patient outpatient visit, patients may present with several health issues that are not captured in our HIV-related versus non-HIV related dichotomy. Fourth, in Canada, family physicians are primary care providers whereas internal medicine physicians act as consultant specialists. This differs from the United States where general medicine specialists may also provide primary care. However, the analysis of ambulatory visits by both physician specialty and HIV caseload allows generalization across settings. Finally, these data do not allow us a deeper understanding of how different specialties share care for the same patients.

## Conclusions

Our study demonstrates that differences exist between specialists caring for people with HIV in Ontario. Further, the distribution of visits unveils patterns of health care delivery by physicians depending on their specialty and their HIV caseloads. Family physicians with high and low caseloads provide substantial proportions of care for this population, as do psychiatrists. Further research should delineate the ways these specialty groups share care for this complex, chronic population and how different provider patterns relate to quality of care.

## Abbreviations

HIV: Human immunodeficiency syndrome
ART: Antiretroviral therapy
ICES: Institute for Clinical Evaluative Sciences
RPDB: Registered Persons Database
OHIP: Ontario Health Insurance Program
IPDB: ICES Physician Database
CPDB: Corporate Provider Database
OPHRDC: Ontario Physician Human Resource Data Centre
SAS: Statistical analysis system

## References

[1] Sackoff JE, Hanna DB, Pfeiffer MR, Torian LV (2006). Causes of death among persons with AIDS in the era of highly active antiretroviral therapy: New York City. Ann Intern Med.

[2] Kitahata MM, Gange SJ, Abraham AG, Merriman B, Saag MS, Justice AC (2009). Effect of early versus deferred antiretroviral therapy for HIV on survival. N Engl J Med.

[3] Hasse B, Ledergerber B, Furrer H, Battegay M, Hirschel B, Cavassini M (2011). Morbidity and aging in HIV-infected persons: the Swiss HIV cohort study. Clin Infect Dis.

[4] Guaraldi G, Orlando G, Zona S, Menozzi M, Carli F, Garlassi E (2011). Premature age-related comorbidities among HIV-infected persons compared with the general population. Clin Infect Dis.

[5] Kendall C, Wong J, Taljaard M, Glazier RH, Hogg W, Younger J (2014). A cross-sectional, population-based study measuring comorbidity among people living with HIV in Ontario. BMC Public Health.

[6] Gallant JE, Adimora AA, Carmichael JK, Horberg M, Kitahata M, Quinlivan EB (2011). Essential components of effective HIV care: a policy paper of the HIV medicine association of the infectious diseases society of America and the Ryan White medical providers coalition. Clin Infect Dis.

[7] Maldonado V. The State of HIV primary care: a shifting landscape. HealthHIV: accessed March 18, 2013.

[8] Rackal JM, Tynan AM, Handford CD, Rzeznikiewiz D, Agha A, Glazier RH (2011). Provider training and experience for people living with HIV/AIDS. Cochrane Database Syst Rev.

[9] Handford CD, Rackal JM, Tynan A-MM, Rzeznikiewiz D, Glazier RH (2012). The association of hospital, clinic and provider volume with HIV/AIDS care and mortality: systematic review and meta-analysis. AIDS Care.

[10] Handford C, Tynan A-M, Rackal J, Glazier R (2006). Setting and organization of care for persons living with HIV / AIDS. Cochrane Database Syst Rev.

[11] Fultz SL, Goulet JL, Weissman S, Rimland D, Leaf D, Gibert C (2005). Differences between infectious diseases-certified physicians and general medicine-certified physicians in the level of comfort with providing primary care to patients. Clin Infect Dis.

[12] Duffus WA, Barragan M, Metsch L, Krawczyk CS, Loughlin AM, Gardner LI (2003). Effect of physician specialty on counseling practices and medical referral patterns among physicians caring for disadvantaged human immunodeficiency virus-infected populations. Clin Infect Dis.

[13] Sheth A, Moore R, Gebo K (2006). Provision of general and HIV-specific health maintenance in middle aged and older patients in an urban HIV clinic. AIDS Patient Care STDS.

[14] Reinhold J-PP, Moon M, Tenner CT, Poles MA, Bini EJ (2005). Colorectal cancer screening in HIV-infected patients 50 years of age and older: missed opportunities for prevention. Am J Gastroenterol.

[15] Leece P, Kendall C, Touchie C, Pottie K, Angel JB, Jaffey J (2010). Cervical cancer screening among HIV-positive women. Retrospective cohort study from a tertiary care HIV clinic. Can Fam Physician.

[16] Maldonado V. HIV Caseload Increasing Among Primary Care Providers. HealthHIV: accessed March 18, 2013.

[17] Chu C, Selwyn PA (2011). An epidemic in evolution: the need for new models of HIV care in the chronic disease era. J Urban Health.

[18] Kendall CE, Guenter D (2006). Time to deliver on HIV care. Can Fam Physician.

[19] Guenter D, Scott S (2004). Short Report: Canadian family doctors caring for people with HIV and AIDS. Can Fam Physician.

[20] Antoniou T, Zagorski B, Loutfy MR, Strike C, Glazier RH (2011). Validation of case-finding algorithms derived from administrative data for identifying adults living with human immunodeficiency virus infection. PLoS One.

[21] Kralj B (2000). Measuring “rurality” for purposes of health care planning: an empirical measure for Ontario. Ont Med Rev.

[22] Mangin D, Heath I, Jamoulle M (2012). Beyond diagnosis: rising to the multimorbidity challenge. BMJ.

[23] Engsig FN, Hansen A-BE, Gerstoft J, Kronborg G, Larsen CS, Obel N (2010). Inpatient admissions and outpatient visits in persons with and without HIV infection in Denmark, 1995-2007. AIDS.

[24] Mugavero MJ, Norton WE, Saag MS (2011). Health care system and policy factors influencing engagement in HIV medical care: piecing together the fragments of a fractured health care delivery system. Clin Infect Dis.

[25] Public Health Agency of Canada. HIV/AIDS Epi Update: National HIV Prevalence and Incidence Estimates in Canada for 2008. Surveillance and Risk Assessment Division, Centre for Communicable Diseases and Infection Control; 2010:1–7

[26] Cunningham WE, Drainoni M, Cunningham CO, Eldred L, Wong MD (2006). Health services utilization for people with HIV infection. Med Care.

[27] Glazier R, Zagorski B, Rayner J (2012). Comparison of Primary Care Models in Ontario by Demographics, Case Mix and Emergency Department Use, 2008/09 to 2009/10. ICES Investigative Report. Toronto, Ontario: ICES Investigative Report.

